# Loss of the puromycin-sensitive aminopeptidase, PAM-1, triggers the spindle assembly checkpoint during the first mitotic division in *Caenorhabditis elegans*

**DOI:** 10.17912/micropub.biology.001167

**Published:** 2024-04-02

**Authors:** Aidan Durkan, Annalise Koup, Sarah E. Bell, Rebecca Lyczak

**Affiliations:** 1 Biology, Ursinus College, Collegeville, Pennsylvania, United States

## Abstract

Puromycin-sensitive aminopeptidases have long been implicated in cell-cycle regulation, but the mechanism remains unknown. Here we show that mutations in the gene encoding the *C. elegans* puromycin-sensitive aminopeptidase, PAM-1 , cause chromosome segregation defects and an elongated mitosis in the one-cell embryo. Depleting a known regulator of the spindle assembly checkpoint (SAC), MDF-2 (MAD2 in humans), restores normal mitotic timing to *pam-1* mutants but exacerbates the chromosome segregation defects. Thus, PAM-1 is required for proper attachment of chromosomes to the mitotic spindle and its absence triggers the SAC.

**Figure 1. Mitotic timing differences in pam-1 mutants due to triggering the SAC:**
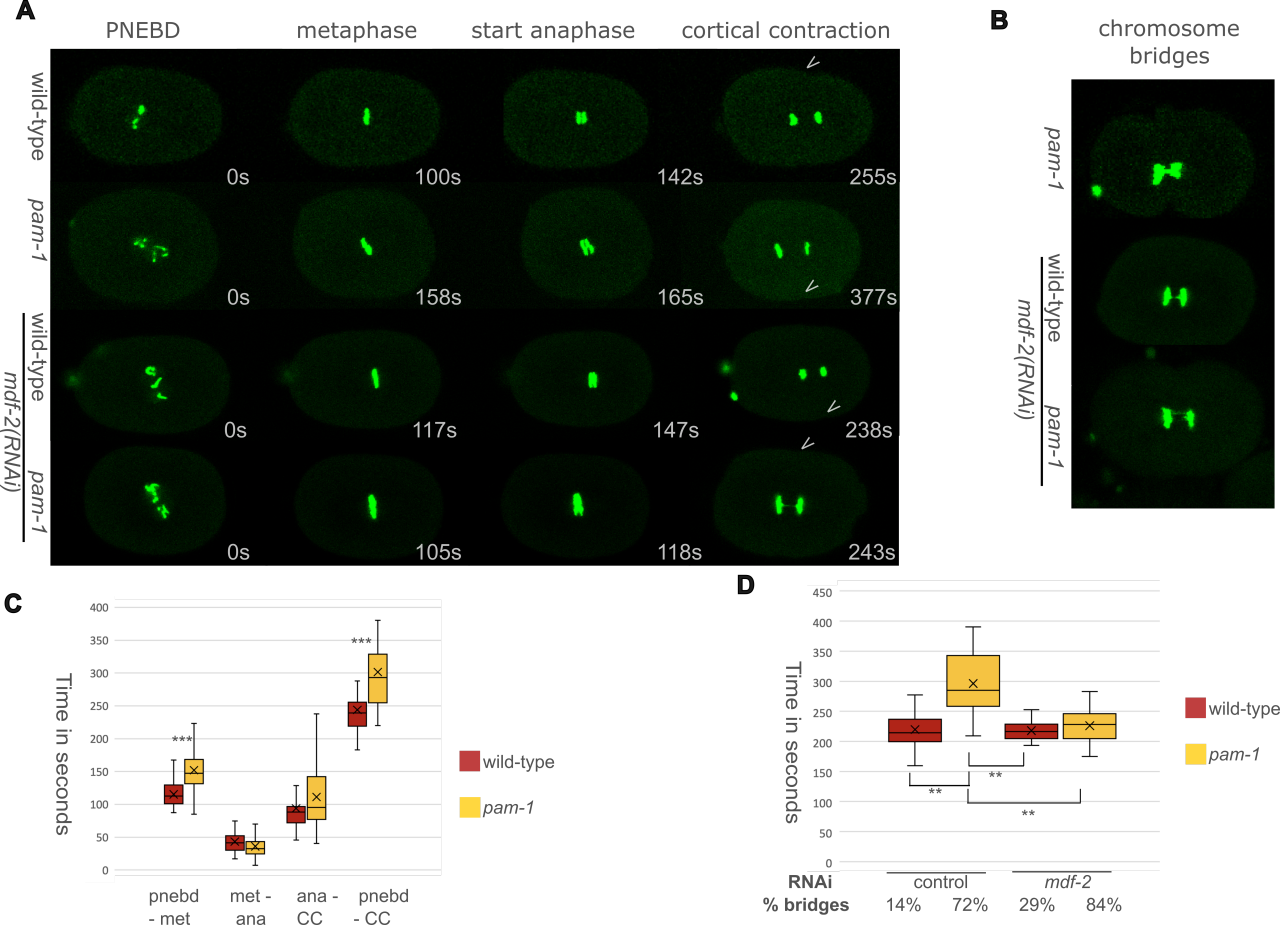
A) Representative images from time-lapse sequences of embryos with GFP tagged histones to view the timings and phases of mitosis. The timing relative to pronuclear envelope breakdown (PNEBD) is depicted for each embryo. The arrowhead demarcates the beginning of cortical contraction of cytokinesis. B) Examples of chromosome segregation defects observed. C) Comparison of the timing of wild-type and *pam-1 ( or403 )* strains for different mitotic phases. A student T-test was used, and significance was determined at p<0.05. *** p<0.0001. (n=37-40 for each strain and phase) D) A comparison of the wild-type and *pam-1 ( or403 )* strains treated with control (L4440) or *mdf-2 (RNAi).* ANOVA for all p< 0.0001 Tukey HSD **p<0.01. for *pam-1 ( or403 )* control as compared to each of the other strains and treatments. In all graphs, error is shown in quartiles, median is marked with a line and mean with an X. Percentage of embryos that exhibit chromosome bridges during anaphase is noted. (n= 28, 28, 23, 25 for each treatment)

## Description

PAM-1 is a conserved puromycin-sensitive aminopeptidase in *C. elegans* (Brooks et al. 2003) *.* In many species, these aminopeptidases have been linked to cell cycle regulation (reviewed in Peer 2011). In *C. elegans,* we have previously found PAM-1 is required for many processes in the early embryo, including timely meiotic exit completion, positioning of the centrosome during polarity establishment, and chromosome segregation (Lyczak et al. 2006; Fortin et al. 2010; Saturno et al. 2017) . Due to these numerous defects, mutations in *pam-1* are maternal-effect embryonic lethal with less than 15% of embryos hatching from *pam-1* mothers (Lyczak et al. 2006) . Considering the mitotic defects observed when puromycin-sensitive aminopeptidases are disrupted in many species (Constam et al. 1995; Huber and O'Day 2011; Huber et al. 2013), we sought to further examine the first mitosis in *pam-1* mutant *C. elegans* embryos *.*

Using GFP-tagged histones, we were able to document the first mitotic division as well as time the phases of mitosis in wild-type and *pam-1* mutant one-cell embryos ( Figure 1 ). We timed from pronuclear envelope breakdown (PNEBD), the time when we could no longer see exclusion of cytoplasmic GFP signal in the nucleus, to the onset of cortical contraction (CC), when a first pinching of the membrane at the start of cytokinesis was observed ( Figure 1A ). We opted to use cortical contraction instead of chromosome decondensation, as it has previously been shown that decondensation is difficult to score in embryos with chromosome segregation defects (Essex et al. 2009) . Overall, we found that *pam-1* embryos take significantly longer to complete mitosis. While wild-type embryos take about 244 seconds to complete mitosis, *pam-1* embryos take 302 seconds on average ( Figure 1C ). In addition, many *pam-1* embryos exhibit chromosome segregation defects ( Figure 1B ). While, we never observed DNA bridges in wild-type embryos, we observed that 23% of *pam-1* embryos had DNA bridges. Additionally, 28% of the *pam-1* embryos had lobed or malformed nuclei at the two-cell stage and difficulty decondensing the chromosomes, additional evidence of chromosome segregation defects. In addition to an effect of chromosome segregation problems, the decondensation difficulty may be similar to what is observed following meiosis in *pam-1* mutants, where delayed decondensation of the chromosomes and meiotic exit have been documented (Lyczak et al. 2006) .

To see if a particular stage of mitosis accounts for the increased length, we separated mitosis into different phases and compared the timings. In addition to PNEBD to start timing and CC to end timing, we looked at metaphase plate formation and the first sign of separation in anaphase. We observed significant differences in the timing of the beginning of mitosis, with *pam-1* embryos taking significantly longer to align chromosomes on the metaphase plate as compared to wild-type. While wild-type embryos align 115 seconds after PNEBD, *pam-1* embryos take 152 seconds to align ( Figure 1C ). Once aligned on the metaphase plate, both strains advanced to anaphase with similar timings. While *pam-1* embryos were overall more variable than wild-type in moving from anaphase to the onset of cortical contraction, there was no significant difference in the timing of this phase of mitosis between the two strains ( Figure 1C ). Thus, *pam-1* mutants take longer to align their chromosomes at metaphase, and this significantly increases the time to complete mitosis.

The spindle assembly checkpoint (SAC) is triggered when kinetochores are not attached to the bipolar spindle microtubules for alignment at metaphase (reviewed in Pintard and Bowerman 2019). MDF-2 is the MAD2 homolog, which localizes to kinetochores that are not yet attached to the spindle (Kitagawa and Rose 1999; Essex et al. 2009; Lara-Gonzalez et al. 2021) . Moreover, the SAC regulates the anaphase-promoting complex APC/C, by inhibiting FZY-1 , the CDC-20 homolog which is required for its activation (Nilsson et al. 2008) . APC/C is necessary for sister chromatid separation by degrading securin (reviewed in Pintard and Bowerman 2019). The SAC's inhibition of the APC/C then delays the onset of anaphase to prevent genetic damage to the cell.

Due to the chromosome segregation defects we observed in *pam-1* embryos, we speculated that the delay in metaphase alignment could be due to a triggering of the spindle assembly checkpoint. If this is the case, depletion of components of the checkpoint machinery, such as MDF-2 , should reduce the timing of mitosis in *pam-1* mutants while increasing the number of chromosome segregation defects. This is indeed what we saw. When we depleted *mdf-2* through RNAi by feeding *,* both wild-type and *pam-1* embryos exhibited a significant increase in the number of chromosome segregation defects due to the lack of spindle assembly checkpoint necessary to ensure proper chromosome attachment to the spindles ( Figure 1B and 1D). Temperature also affected the formation of DNA bridges in the strains, as control RNAi done at 25°C increased the appearance of chromosome bridges in wild-type to 14% and in *pam-1* to 72%. However, when *mdf-2* was depleted by RNAi, both wild-type and *pam-1* embryos exhibited more chromosome segregation defects. Wild-type embryos treated with *mdf-2 (RNAi)* exhibited DNA bridges 29% of the time, while *pam-1 ; mdf-2 (RNAi)* embryos exhibited DNA bridges 84% of the time, suggesting that in *pam-1* mutants, the SAC is allowing some *pam-1* embryos to successfully segregate their chromosomes. Prior work on depletion of SAC components such as *mdf-1 , mdf-2 ,* or *san-1 /mdf-3* also showed DNA segregation errors, although this was always more prominent when the cells were stressed by anoxia or spindle defects (Encalada et al. 2005; Hajeri et al. 2005; Stein et al. 2007; Hajeri et al. 2008) .

In prior work, when SAC components were depleted in cells with kinetochore attachment problems, mitotic timing was returned to normal (Encalada et al. 2005; Essex et al. 2009) . We observed the same here as both wild-type and *pam-1* embryos showed similar mitosis completion timings when *mdf-2* was inactivated ( Figure 1D ). There was no significant difference in the timing of mitosis in wild-type strains with control or *mdf-2 (RNAi)* or *pam-1 ; mdf-2 (RNAi)* embryos ( Figure 1D ). This confirmed that the increased mitotic timing in *pam-1* embryos is due primarily to a failure to properly attach the chromosomes to the spindle and suggests that PAM-1 is required and/or plays a critical role for this process.

PAM-1 is a cytoplasmic aminopeptidase with few known targets identified. At mitosis, PAM-1 is seen to concentrate around the mitotic spindle and chromosomes, suggesting it may act to regulate chromosome attachment to the spindles (Fortin et al. 2010) . Interestingly, MDF-2 is localized similarly during meiosis and mitosis (Kitagawa and Rose 1999) . In addition to the chromosome segregation defects in mitosis, we previously found a similar defect in meiosis II, but not meiosis I, suggesting that PAM-1 may only be required for sister chromatid attachment to the spindles and their separation (Lyczak et al. 2006) . *pam-1* mutants also have meiotic exit defects with a failure the chromosomes to decondense appropriately after meiosis II, a defect that was rescued by depletion of cyclin B3, *cyb-3* (Lyczak et al. 2006) . As, we also saw decondensation problems during mitosis, this may be something to examine further. Cyclin B3 levels can also affect the timing of anaphase entry by regulating the SAC (Tarailo-Graovac and Chen 2012) , further suggesting that PAM-1 may be influencing the SAC through CYB-3 .

Another interaction to explore is one with WEE-1.3 . In our previous work, we found that a mutation in *wee-1.3 ,* which encodes a kinase which negatively regulates CDK-1 , suppresses some *pam-* 1 phenotypes (Benton et al. 2021) . As CDK-1 phosphorylates FZY-1 and the APC/C (reviewed in Pintard and Bowerman 2019), and the APC/C has been shown to associate with the cyclinB3/CDK complex, and WEE1 (Vassilopoulos et al. 2015; Garrido et al. 2020) , it will be interesting in the future to explore if the genetic interaction between *pam-1* and *wee-1.3* are involved in the mitotic defects in *pam-1* mutants. Future work should focus on potential targets of PAM-1 at the kinetochore or spindle.

## Methods

Strains were maintained at 15°C as described (Brenner 1974) . Embryos were released on a coverslip and imaged on a 3% agarose pad. Time-lapse images were taken on a Nikon EZ-C3 confocal with NIS software every 15 seconds in 5 Z-steps of 1 microns. Set landmarks of mitosis were scored to determine timings. Differences in timings were analyzed by ANOVA or student TTEST. RNAi experiments were as described in (Kamath and Ahringer 2003) and used the feeding vectors, L4440 as a control and Y69A2A_2326.a for *mdf-2 .* Worms were placed on feeding plates at the L4 stage and imaged after treatment for 24 hours at 25°C.

## Reagents

**Table:** 

Strain	genotype	Available from
SX1287	*mjIs145 II; unc-119 ( ed3 ) III *	CGC (Bagijn et al. 2012)
US158	*mjIs145 II; pam-1 ( or403 ) IV *	Lyczak lab
